# Horizontal Ridge Augmentation: A Comparison between Khoury and Urban Technique

**DOI:** 10.3390/biology10080749

**Published:** 2021-08-05

**Authors:** Javier Sánchez-Sánchez, Finn Niclas Pickert, Luis Sánchez-Labrador, Francisco GF Tresguerres, José María Martínez-González, Cristina Meniz-García

**Affiliations:** Department of Dental Clinical Specialties, Faculty of Dentistry, Complutense University of Madrid, 28040 Madrid, Spain; fipicker@ucm.es (F.N.P.); luissanc@ucm.es (L.S.-L.); frango09@ucm.es (F.G.T.); jmargo@odon.ucm.es (J.M.M.-G.); cmenizga@ucm.es (C.M.-G.)

**Keywords:** dental implants, guided bone regeneration, horizontal bone augmentation, bone graft

## Abstract

**Simple Summary:**

With the emergence of novelty regeneration techniques in the implant dentistry field, the professional may have some queries about which one to use in their daily practice. No systematic review, to date, analyzes the horizontal gains achievable with the two main procedures for bone regeneration: the Khoury technique, which uses split blocks obtained from the patient himself, or the Urban technique, which employs membranes to contain the biomaterials. Regarding this paper, the reader will be able to decide if any of these options is adequate for the indication required.

**Abstract:**

Purpose. The objective of this systematic review was to evaluate and compare the clinical efficacy of horizontal alveolar ridge augmentation techniques described by Khoury and Urban. Methods. A systematic electronic search in the MEDLINE databases, SCOPUS, WOS, and the Cochrane Central Register of Controlled Trials (CENTRAL) as well as a manual search, were conducted independently by two reviewers up to July 2021. Results. Six studies met the pre-established inclusion criteria and were included in the descriptive analysis. Due to the heterogeneity found across the included studies, meta-analysis could not be performed. Horizontal bone gain was between 3.93 ± 0.9 mm and 5.02 ± 0.8 mm with the Khoury technique and between 3.9 ± 0.9 mm and 5.68 ± 1.42 mm with the Urban technique. Similar complication rates were reported in both groups: infection (7%), in the Khoury technique, and membrane exposure (3.2–13.6%), in the Urban technique, being the most frequent events. Conclusions. Both techniques were found to be effective, in terms of clinical bone gain, for horizontal alveolar ridge gain. Nevertheless, available literature is limited, and there is a lack of comparative studies to better evaluate the results.

## 1. Introduction

Tooth loss is accompanied by a series of adaptive changes, leading to dimensional alterations of the alveolar process. During the first year after tooth extraction, Schropp et al. [1] described 50% bone loss in the buccal-lingual dimension, with 30% of the initial ridge width lost during the first 3 months. In addition, this dimension reduction of the jaws also affects vertically. Over time, these resulting ridge deficiencies may limit proper dental implant placement [2,3,4,5].

In day-to-day practice, it is common to deal with insufficient alveolar crests that may require surgical augmentation for placing implants in a correct position. Monje et al. [6] showed, in a recent preclinical study, that implants placed with a sufficient peri-implant buccal bone wall thickness, greater than 1.5 mm, were exposed to significantly less bone loss compared to those placed in sites exhibiting less than 1.5 mm. Therefore, the presence of alveolar ridge deficiencies often requires reconstructive procedures in order to increase the alveolar ridge to allow correct implant placement. Different procedures have been described, such as short and narrow implants to avoid bone regeneration or techniques to regenerate, in a horizontal and vertical way, the alveolar crests, distraction osteogenesis, guided bone regeneration, or block grafts, to obtain an adequate alveolar dimension. Bone augmentation is justified in three circumstances: in cases where an implant cannot be placed in its ideal position, its long-term success or primary stability is abridged, or if high aesthetic requirements are demanded [7,8,9,10,11,12].

In every regenerative procedure, it is mandatory to accomplish four major principles that Wang et al. [13] described as PASS and allow a more predictable GBR: primary closure to facilitate a protected environment from microbiota and mechanical forces, angiogenesis to promote de novo bone formation, space to assure the different compartments and avoid the collapse of the biomaterial, and stability of the blood clot. These requirements enable better results and reduce the incidence of possible adverse effects.

Within the wide range of different augmentation procedures, guided bone regeneration (GBR) and the use of autogenous bone blocks are the most common interventions in bone augmentation; Khoury et al. [14] described the stabilization of two split autologous bone blocks by microscrews and filling the generated gap with autogenous bone chips. These split bone blocks are obtained, either from the mandibular symphysis or ramus, using piezoelectric surgery or microsaws, obtaining a block that will later be divided into the two final thin laminae. Among the great variety of different GBR procedures for horizontal bone augmentation, Urban et al. [15,16] described the utilization of a 1:1 mixture of autogenous and xenogenic graft material, covered by a resorbable collagen membrane that is stabilized by titanium pins.

Due to the limited comparative information about these two techniques specifically, without combining them with other procedures or materials, this systematic review aims to evaluate and compare horizontal clinical bone gain and complications arising from the alveolar ridge augmentation techniques described originally by Khoury and Urban in the treatment of horizontal ridge deficiencies.

## 2. Materials and Methods

### 2.1. Protocol

The guidelines of the Preferred Reporting Items for Systematic Reviews and Meta-Analyses statement (PRISMA) were followed in this systematic review [17]. It was registered in the International Prospective Register of Systematic Reviews (PROSPERO) (Reg. No. CRD42021229169).

The following focus question was developed according to the PICOS design (Population, Intervention, Comparison, Outcomes and Study type) (Table 1).

Question of interest: “In patients with horizontal alveolar ridge deficiencies (Population), what is the horizontal ridge augmentation obtained (Intervention) by means of the Khoury and Urban techniques (Comparison) in terms of clinical horizontal bone gain (Outcome) found in RCTs and case series (Study type)?”

### 2.2. Eligibility Criteria

#### 2.2.1. Inclusion Criteria

Studies were included when they met the following inclusion criteria:

1. Including at least 10 adult patients without contraindication to oral surgery who presented horizontal alveolar ridge defects requiring reconstructive procedures aimed at augmenting the alveolar ridge to allow proper placement of dental implants;
2. Treated by either the Khoury or the Urban technique;
3. Studies reporting an observation period of at least 4 months, analyzing volumetric changes to the alveolar ridge.

#### 2.2.2. Exclusion Criteria

1. Editorials, reviews, preclinical studies, animal studies, in vitro studies;
2. Studies reporting simultaneous implant placement;
3. Studies evaluating onlay block grafts placed in direct contact to the alveolar ridge or studies describing the additional use of other biomaterials or bioactive substances in combination with the above defined techniques.

### 2.3. Search

A systematic electronic search in the MEDLINE databases, Cochrane Central Register of Controlled Trials (CENTRAL), SCOPUS, and WOS was performed up to July 2021 by applying the following combination of keywords:

((((autologous) OR (autogenic)) OR (autogenous)) AND (((bone block) OR (split bone block)) OR (block graft))) OR (((((xenogenic) OR (bovine)) OR (xenograft)) AND ((guided bone regeneration) OR (GBR))).

Two reviewers (J.S.S, F.P) independently performed a two-step screening process. At Stage 1, the investigators independently screened all titles and abstracts found by the electronic search for relevance. Controversial articles were included for additional evaluation during the following stages in cases of uncertainty. During Stage 2, full-texts of all pre-selected articles were independently reviewed by the investigators to further exclude articles that did not meet the predetermined inclusion criteria, and a third investigator (L.S.L) was consulted in cases of disagreement. Then, an additional manual search was performed based on the references from the definitive list of included articles.

### 2.4. Data Collection

The following qualitative and quantitative data from the included studies were collected:

1. General study, characteristics, and demographic data of subjects (author, year of publication, number of groups studied, number of subjects in each group, method of measurement of variables, age and gender distribution of subjects, history of smoking habits);
2. Surgical procedures (defect localization, graft material and membrane, donor site, method to obtain the graft, post-surgical pharmacological treatment);
3. Qualitative data for the assessment of possible risk of bias;
4. Outcome variables of interest.

The outcome variables were classified according to the temporal sequence of the augmentation procedures: T^0^ represented the moment when grafting procedures were performed, T^1^ represented the evaluation of the augmented site when implants were placed, and T^2^ represented the latest follow-up data. At T^0^, defect size and graft dimensions were registered. At T^1^, clinical bone gain (CBG), graft resorption (R), and histological data (H) were registered. At T^2^, implant survival (IS) and marginal bone loss (MBL) were analyzed.

### 2.5. Quality Assessment

The assessment of possible risk of bias for the included clinical trials was performed according to the *Cochrane* guidelines and, for the included case series, according to the Joanna Briggs guideline [18,19].

### 2.6. Descriptive Analysis

The collected data was organized into tables and analyzed in order to evaluate similarity and differences between the two augmentation procedures described by Khoury and Urban. The data analyzed comprised: clinical bone gain, implant survival rates, marginal bone loss, graft resorption, complication rates, and histological variables.

## 3. Results

### 3.1. Study Selection

**Figure 1:**
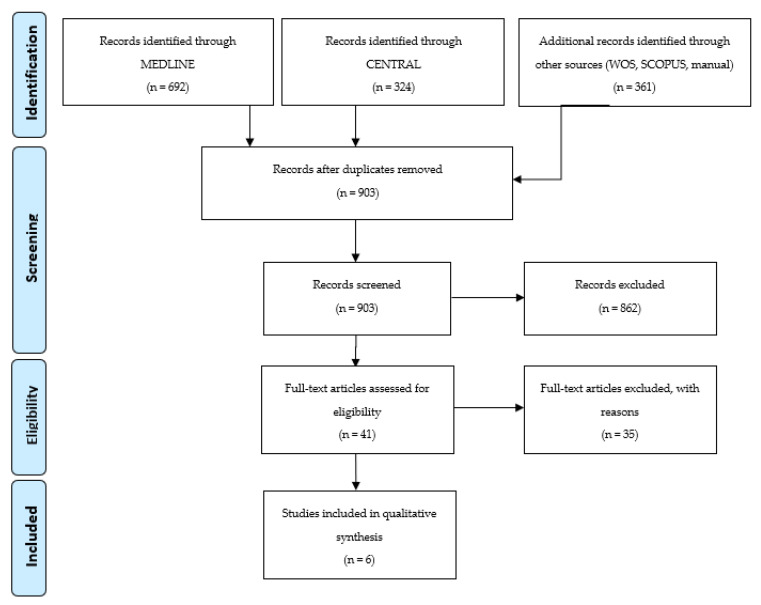
Prisma Flow Diagram.

A total of 1377 studies were identified through database searches, and 4 additional articles were found by manual searches. After deleting duplicate studies, 903 studies were obtained. Following title and abstract screening, 862 studies were excluded and 41 studies were included for full-text assessment. Thirty-five additional records were excluded, resulting in 6 final studies that met the eligibility criteria and were included for descriptive analysis. Due to the great heterogeneity found across the included studies, meta-analysis could not be performed. The list of articles excluded from this review and the reasons are presented in supplementary Table S1. Figure 1 shows a flow diagram of the search results.

### 3.2. Study Characteristics

A detailed overview of patients and studies characteristics is shown in Table 2. Included studies were three case series [16,20,21] and one clinical trial [22] concerning the Urban technique and two randomized clinical trials [23,24] concerning the Khoury technique. There were no comparative studies. The included studies were published between 2013 and 2020. Furthermore, two studies [16,20] reported the exclusion of patients with smoking habits, one study reported ten smokers and eight non-smokers [21], and the remaining three studies did not report information regarding smoking habits. In all studies analyzing the Khoury technique, the bone grafts were obtained from the mandibular symphysis or the ramus of the mandible. In all studies except one [16], amoxicillin or amoxicillin/clavulanic acid were prescribed following the interventions, and in two studies just ibuprofen was prescribed [16,21].

All studies recorded measurements from CBCT except the study by Urban et al. [16], which used a caliper measuring 2 mm apically from the top of the crest and periapical radiographs. Follow-up was 4 months in the Khoury studies, while it was 6–8 months in studies that analyzed the Urban technique.

### 3.3. Risk of Bias Assessment

For the overall risk-of-bias judgment of the included clinical trials, one study was assessed to be at low risk of bias and two of moderate risk of bias. For the overall risk-of-bias judgment of the included case series, one study was assessed to be at low risk of bias and two of moderate risk of bias [18,19]. (Tables S2 and S3).

### 3.4. Outcome Variables

Primary outcomes are summarized in Table 3.

#### 3.4.1. Clinical Bone Gain (CBG)

Initial horizontal ridge width (T^0^) in studies analyzing the GBR technique described by Urban [14,18,19,20] ranged between 2.19 ± 0.64 mm and 4.47 ± 0.25 mm, while in the two studies analyzing the Khoury technique [23,24], the initial ridge width was found to be 2.67 ± 0.61 and 3.85 ± 0.6. At the time of implant placement (T^1^), it was 3.9–5 mm in the Khoury group and between 1.41–5.6 mm in the Urban group. CBG was between 3.93 ± 0.9 mm and 5.02 ± 0.8 mm in the Khoury studies and between 1.41 ± 0.08 mm and 5.68 ± 1.42 mm in the Urban studies.

#### 3.4.2. Graft Resorption (R)

Three studies reported information on graft resorption between T^0^ and T^1^ [22,23,24]. One study evaluating the GBR technique described by Urban [21] noticed 0.6 mm of resorption after 6 months. In the studies analyzing the Khoury technique [23,24], Bartols et al. reported 2.3 mm of resorption after 12 months and Atef et al. [23] observed 0.36 mm resorption after 4 months.

#### 3.4.3. Complications (COM)

From the GBR studies, two of them, Urban et al. and Atef et al. [16,22], reported cases of infection (3.2% and 10% of the patients, respectively), but both cases had an uneventful healing. Meloni et al. [21] observed three cases of membrane exposure (13.6% of the patients) that did not affect the planned treatment and were solved with antiseptic applications. Saravanan et al. [20] did not report any information of possible complications.

One study evaluating the Khoury technique, Bartols et al. [24], reported the complete loss of one bone graft due to an infection (7% of the grafts), and Atef et al. [23] observed one case of a temporary paresthesia that resolved spontaneously after one month.

#### 3.4.4. Histomorphometric Analysis (H)

Only two studies [16,22] that evaluated the GBR technique described by Urban reported data from a histomorphometric analysis. Urban et al. [16] reported 31% of regenerated bone, 25.8% of remaining graft particles, and 43.2% of marrow spaces at a mean of 8.4 months after intervention, while Atef et al. [22] found 28% of regenerated bone, 23.78% of remaining graft particles, and 48.11% of marrow spaces, after 6 months of healing. No histomorphometric data is contributed by Khoury’s studies.

#### 3.4.5. Implant Survival (IS)

Two studies evaluating the GBR technique described by Urban [16,21] and one study evaluating the Khoury technique [24] reported 100% on implant survival at T^2^, which ranged between 1–3 years; 20.88 ± 9.49 months in the study by Urban et al. [16], 1 year in the study by Bartols et al. [24], and 3 years in the study by Meloni et al. [21].

#### 3.4.6. Marginal Bone Loss (MBL)

One study assessing the Khoury technique, Bartols et al. [24], described a marginal bone loss of 1.9 ± 2.86 mm after 1 year of implant insertion, measuring a CBCT at the mesial and distal margin of the implant shoulder similar to a 2D radiographic assessment, and one study evaluating the GBR technique, Meloni et al. [21], reported a marginal bone loss of 1.03 ± 0.21 mm after 1 year and 1.15 ± 0.28 mm after 3 years, evaluated with peri-apical x-ray.

## 4. Discussion

In this systematic review, the clinical efficacy of two horizontal bone augmentation techniques, as described by Khoury and Urban, were evaluated [16,25]. Both have been shown to be effective in achieving clinical augmentation of the alveolar ridge in horizontal bone defects, showing similar results, with bone gains of up to 5.6 mm in the “Sausage” technique [16,20,21,22] and up to 5 mm in the Khoury technique [23,24]. Saravanan et al. [20] only showed a horizontal bone gain of 1.41–1.44 mm compared to the rest of the studies evaluating the Urban technique [16,21,22], which obtained a bone gain of 3.9 mm −5.6 mm. According to the same study, when performing the horizontal regeneration in very favorable defects (Seibert type I) in anterior teeth, a big augmentation is not mandatory to place the implants, hence their low bone gain.

Three studies recorded resorption measures [22,23,24]. Grafts undergo large resorption differences depending on the period in which they are observed. However, as these are not perfectly comparable studies (neither in patients nor observation periods), and even mixing the two techniques, they are not completely comparable results. Besides, the studies included in this review have short follow-up times, so the results about marginal bone loss should be interpreted with caution. All these observations were taken from the measurements by means of CBCT, except in one study, Urban et al. [14], which used a caliper to measure the crest width. Scientific evidence shows that differences between both methods are minimal: Banodkar et al. [26] showed a high correlation between intrasurgical measures and CBCT ones of 0.988 (98.80%), obtaining more exact results in horizontal defects as compared to vertical defects. Along the same line, Mehra et al. [27] did not report significant differences measuring with CBCT, being of about −1.59% compared to direct observation. Nowadays, evaluation with calipers to determine ridge dimensions is not the most appropriate method to determine horizontal bone gain.

Different resorption rates are described in the literature depending on the type of material employed, being higher in autologous bone than in xenograft. In line with the existing evidence, resorption in xenograft cases is very unpredictable; however, these particles are intimately united in a dense network of newly formed tissue. Von Arx et al. [28] covered the autogenous bone block with a xenograft layer and a collagen membrane. At re-entry, a fibrous encapsulation of the xenograft was seen; a big difference was seen when using a mixture of both grafts together. In the study by Mordenfeld et al. [29], they studied bone gain with different proportions of auto-xeno; they compared 90:10 to 60:40. Bone gain was similar, but there was significantly less resorption in the 60:40 case, which could be explained by a higher resorption rate of autologous bone [28,30,31]. Creeping substitution could explain this greater resorption due to growth factors provided by autologous bone because it presents vital osteocytes, which are responsible for mechanotransduction and can promote bone resorption secondary to mechanical stimulus, especially when bone augmentation goes beyond the bony framework of the alveolar process. As scientific evidence suggests, resorption in these cases is very unpredictable and, in this review, only one study was found to indicate graft resorption [23], with a loss of 4% at 4 months, without being statistically significant and not being possible to compare both techniques [32,33,34].

Studies in which a membrane was added over the autologous block graft have been excluded, but the literature shows lower levels of resorption when attaching membranes or bone substitutes. Many factors could affect the graft if a barrier is not placed, such as lack of cell exclusion or greater impairment of muscle action [35,36]. This different resorption rate could be explained by the mechanism of the Moss “functional matrix”, which proposes that bone maintenance depends on compensatory and mechanically responses. In this sense, it is suggested that bone is delimited by soft tissue edges; when a graft is placed it disrupts this natural soft tissue boundary, resulting in an increase of the muscular forces in that area, promoting bone resorption as a homeostatic response [28,29,30,31].

Although autogenous bone has been described by some authors as the ‘*gold standard*’ for bone augmentation procedures due to its osteogenic, osteoinductive, and osteoconductive properties, it suffers several disadvantages, such as higher morbidity, the need for a donor site, and the limited quantity of bone available [37]. Besides, autogenous bone block grafting presents a range of complications derived from the technique, occurring in 30–50% of cases [38,39,40]. Of these, the most serious is neurosensory disturbance, often observed in cases of autogenous chin bone harvesting, which can also produce aesthetic changes in the patient’s facial contours [38,41]. Nevertheless, in this systematic review, just one case of paresthesia was described [23].

The use of blocks or split blocks of autologous bone is one of the most common procedures for treating ridge defects. These augmentation techniques have shown an implant survival rate of 95–98% [42,43]. In this review, three studies [16,21,24] showed a 100% implant survival placed in regenerated areas after 1 to 3 years of follow-up, but there are studies with a shorter observation period and a smaller sample size. Nevertheless, these results are similar than those obtained by Pistilli et al. [44], with a 98.77% of survival rate of implants placed in bone regenerated with autogenous bone onlay blocks, compared to 82.8% of survival rate of implants placed in equine bone blocks [37]. In addition, in the systematic review of Sanchez-Labrador et al. [45], a higher survival implant rate was obtained when autogenous bone blocks were used, in comparison to xenogeneic bone blocks.

Regarding histomorphometric analysis of this review, derived from just two studies, Urban et al. and Atef et al. [16,22], it was shown that around 30% of the bone biopsy corresponds to regenerated bone and around a 25% of xenograft remains that has not been able to be remodeled by osteoclasts as they are too dense particles. In Urban et al. [16] the histologic samples were collected at a mean 8.4 s after intervention, and Atef et al. [22] collected the samples after 6 months of healing. Both of them had similar results, not finding differences in healing time. Regarding the evidence concerning dental implants placed in regenerated bone, the type of graft material does not affect the long-term results, according to Nevins et al. [46]. In addition, the combination of xenografts with autogenous bone brings combined benefits as the autogenous bone lacks the structural strength necessary; in these cases, where the maintenance of space is essential, the xenograft particles provide this characteristic. On the other hand, xenogenic bone is not osteogenic nor osteoinductor, a characteristic that is present in the autogenous bone [47,48,49]

It should be considered that the two procedures are very dependent on surgical technique and require a high level of training and clinical experience to achieve good results. With regard to specific indications for each of them, there is no consensus in the existing evidence that indicates scenarios where one or the other are more effective. What is evident is that, whenever autologous bone is obtained, a trauma is caused so, in cases with limited donor sites or potential risk of nerve injury, the Khoury technique should be avoided, and the use of membranes will be the complementary option. On the other hand, Khoury’s technique does not utilize exogenous materials that can induce host responses and affect the results of regenerative act [46,47].

The present study is the first systematic review that compares the clinical findings of two of the most used techniques for bone regeneration in the field of implant dentistry, limiting the influence of confounding factors that may be derived from the combination of these techniques with other biomaterials or membranes. More controlled clinical trials are needed to better evaluate and compare the clinical efficacy of these bone regeneration techniques. Longer follow-up periods are also needed to understand marginal bone loss around implants placed in bone regenerated with both techniques.

## 5. Conclusions

With the limitations of this systematic review, it may be concluded that both techniques, when performed to achieve horizontal bone gain, have been shown to be effective, with similar complication rates and horizontal bone gain.

The availability of RCTs comparing these techniques specifically is scarce, so it will be appropriate to design comparative studies in future in order to understand implant behavior and marginal bone loss with a longer follow-up.

## Figures and Tables

**Table 1:** PICOS question.

PICOS	Description
**Patient (P)**	Patiens with horizontal bone deficiences in the alveolar crest
**Intervention (I)**	Horizontal bone regeneration with the Split bone block technique described by Khoury.
**Comparation (C)**	Horizontal bone regeneration with the Sausage technique desbribed by Urban.
**Outcome (O)**	Clinical and histological efficacy of both techniques Principal effect variable: horizontal bone gain Secondary effect variables: implant survival rates, marginal bone loss, graft resorption, complication rates, and histological variables Randomized Controlled clinical trials (RCTs) and Case series

**Study type (S)**	

**Table 2:** Sample and study characteristics. PCS: Prospective case series; RCT: Randomized controlled clinical trial; GBR: Guided bone regeneration; SBB: Split bone block; Max: Maxilla; Mand: Mandible; Ant: Anterior; Post: Posterior; ABBM: anorganic bovine bone mineral; n/a: Non-applicable. (Grey lines correspond to Urban procedures and white lines to Khoury).

Authors	Year	Study Design	Intervention Test (GBR/SBB)	Intervention Control	Donor Site	Recipient Site	Xenograft	Membrane	Graft Harvesting Technique	Age Range/Mean ± SD	Man/Woman	Tobacco (No Smokers/Smokers)	Antibiotics	NSAIDs
Saravanan P et al.	2013	Prospective case series	GBR	n/a	Symphysis	Max	BioOss	BioMend	Scraper	20–50	n/a	No smokers	Amox 500 mg	Ibuprofen 400 mg
Urban IA et al.	2013	Prospective case series	GBR	n/a	n/a	Post Max + Mand	ABBM	CM	n/a	52.7 ± 11.4	10/15	No smokers	n/a	n/a
Bartols A et al.	2018	Randomized clinical trial	SBB	GBR	External oblique line	Ant Max	n/a	n/a	Disc	19–72/51.5 ± 17.3	7/8	n/a	Amox 750 mg	Ibuprofen 400 mg
Meloni SM et al.	2019	Prospective case series	GBR	n/a	Retromolar	Post Max + Mand	ABBM	CM	Scraper	24–78/56.8	7/11	10/8	Amox 1 g	n/a
Atef et al.	2019	Randomized clinical trial	SBB	SBB onlay block	Symphysis	Post Mand	n/a	n/a	Disc	29–54/42.1	9/11	n/a	Amox/ácido clavulanico 1 g	ibuprofen 600 mg
Atef M et al.	2020	Randomized clinical trial	GBR	GBR with titanium mesh	External oblique line / Symphysis	Max	ABBM	CM	Trephine	n/a	n/a	n/a	Amox/clavulanic acid 1 g	Ibuprofen 600 mg

**Table 3:** Ridge measurements before and after augmentation. NA: Not applicable.

Study	Technique	Width in T = 0	T Observation	Width in T = 1	Horizontal Gain	Resorption
Saravanan 2013	Urban	2 mm: 3.63 ± 0.29 mm	6 months	2 mm: 5.07 ± 0.25 mm	1.44 ± 0.09 mm	NA
		4 mm: 4.1 ± 0.18 mm		4 mm: 5.51 ± 0.21 mm	1.41 ± 0.08 mm	
		6 mm: 4.47 ± 0.25 mm		6 mm: 5.88 ± 0.27 mm	1.41 ± 0.08 mm	
Urban 2013	Urban	2 mm: 2.19 ± 0.64 mm	8.9 ± 2.1 months	2 mm: 7.87 ± 1.61 mm	5.68 ± 1.42 mm	NA
Bartols 2018	Khoury	Pre: 2.67 ± 0.61 mm	12 months	6.60 ± 1.18 mm	3.93 mm	2.33 mm
		Post: 8.93 ± 1.05 mm				
Meloni 2019	Urban	3.07 ± 0.64 mm	7 months	8.09 ± 2.16 mm	5.03 ± 2.15 mm	NA
Atef 2019	Khoury	Pre: 3.85 ± 0.6 mm	4 months	8.84 ± 0.54 mm	5.02 ± 0.8 mm	0.36 mm
		Post: 9.22 ± 0.64 mm				
Atef 2020	Urban	Pre: 3.3 ± 0.4 mm	6 months	7.3 ± 0.9 mm	3.9 ± 0.9 mm	0.6 mm
		Post: 7.9 ± 0.6 mm				

## Data Availability

Data available on request due to restrictions.

## Supplementary Materials

The following are available online at https://www.mdpi.com/article/10.3390/biology10080749/s1, Figure S1: title, Table S1: List of excluded articles and reason for exclusion; Table S2: Cochrane Risk of bias of clinical trials; Table S3: Joanna Briggs Risk of bias of case series.
