# Supplementary material for: Horizontal Ridge Augmentation: A Comparison between Khoury and Urban Technique
Source: Biology (Basel). 2021 Aug 5;10(8):749. doi: 10.3390/biology10080749 (PMC8389589; doi:10.3390/biology10080749)
Supplement: Supplementary file 1 [file biology-10-00749-s001.zip › biology-1294400-supplementary.pdf]

Supplementary Material:

First author and year of publication	Reason for exclusion
Acocella A et al. 2010	Onlay block graft
Alérico FA et al. 2019	Unclear measurement methodology
Amorfini L et al. 2014	Simultaneous implant placement
Cordaro L et al. 2011	Onlay block graft
D'Amato S et al. 2015	Insufficient sample size
De Stavola L et al. 2013	Vertical bone augmentation
Ersanli S et al. 2004	Use of other biomaterials
Ersanli S et al. 2016	Use of other biomaterials
Gulinelli JL et al. 2017	Onlay block graft
Holtzclaw DJ et al. 2010	Split-ridge technique
Jung RE et al. 2009	Simultaneous implant placement
Khoury F et al. 2019	Vertical bone augmentation
Korsch M et al. 2019	Use of other biomaterials
Korzh DG et al. 2019	Full-Text not accessible
Moukrioti J et al. 2019	Unclear measurement methodology
Meloni SM et al. 2017	Sample studied reported in another study
Novy LFS et al. 2019	Vertical bone augmentation
Peñarrocha-Diago M et al. 2013	Use of other biomaterials
Restoy-Lozano A et al. 2015	Vertical bone augmentation
Santana RB et al. 2015	Onlay block graft
Schlee M et al. 2014	Use ot other biomaterials
Schwartz-Arad D et al. 2005	Onlay block graft
Spin-Neto R et al. 2014	Onlay block graft
Spin-Neto R et al. 2013	Onlay block graft
Spin-Neto R et al. 2015	Onlay block graft
Spin-Neto R et al. 2013	Onlay block graft
Thoma DS et al. 2018	Use of other biomaterials
Thoma DS et al. 2019	Use of other biomaterials

Urban IA et al. 2011	Use of other biomaterials
Urban IA et al. 2017	Use of other biomaterials
Verdugo F et al. 2011	Onlay block graft
Verdugo F et al. 2012	Onlay block graft

Table S1 . List of excluded articles and reason for exclusion.

Authors	Year	Study design	Random generation of the secuence	Allocation concealment	Blinding of participants and staff	Blinding of evaluator	Incomplete data	Selective notification of results	Another bias
Bartols A et al.	2018	Clinical trial	Low risk	Low risk	Low risk	Low risk	Low risk	Low risk	Low risk
Atef et al	2019	Clinical trial	Low risk	Not clear risk	Not clear risk	Not clear risk	Low risk	Low risk	Not clear risk
Atef M et al.	2020	Clinical trial	Low risk	Not clear risk	Not clear risk	Not clear risk	Low risk	Low risk	Not clear risk

Table S2. Cochrane Risk of bias of clinical trials.

Authors	Year	Study design	Clear inclusión criteria	Standard and reliable condition measure	Correct condition identification	Consecutive inclusión of participants	Complete inclusión of participants	Complete demographic information	Clinical information	Clear follow up outcomes	Clear reporting of the demographic information of sites/clinic	Statistical analysis appropiate
Saravanan P et al.	2013	Case series	Yes	Yes	Yes	Not clear	Yes	Not clear	Yes	Not clear	Not clear	Yes
Urban IA et al.	2013	Case series	Yes	Yes	Yes	Not clear	Yes	Yes	Yes	Yes	Yes	Yes
Meloni SM et al.	2019	Case Series	Yes	Yes	Yes	Yes	Yes	Yes	Yes	Yes	Yes	Yes

Table S3. Joanna Briggs Risk of bias of case series.
